# Inverted internal limiting membrane flap technique for retinal detachment due to macular holes in high myopia with axial length ≥ 30 mm

**DOI:** 10.1038/s41598-022-08277-y

**Published:** 2022-03-11

**Authors:** Changzhong Xu, Chao Feng, Mengyao Han, Junwen He, Rui Zhang, Tao Yan, Xiangyun Li, Yong Liu, Yanzi Li, Jianhua Wu

**Affiliations:** 1 Department of Ophthalmology, The Second Xiangya Hospital, School of Ophthalmology, Central South University, Changsha, China; 2 Aier Eye Hospital of Wuhan University, No. 481 Zhongshan Road, Wuchang District, Wuhan, China

**Keywords:** Retinal diseases, Vision disorders

## Abstract

To compare the efficacy of internal limiting membrane (ILM) flap covering to that of ILM flap insertion for the treatment of macular hole retinal detachment (MHRD) in highly myopic eyes with axial length (AL) ≥ 30 mm. We retrospectively analysed the medical records of 48 MHRD patients with high myopia (AL ≥ 30 mm). According to different surgical methods, the patients were divided into a covering group (23 eyes) and an insertion group (25 eyes). The rate of retinal reattachment and MH closure were compared between the two groups, and the related factors affecting the initial anatomical results were analysed. After primary vitrectomy and single silicone oil removal, there were 18 eyes (78.3%) in the covering group, and 20 eyes (80.0%) in the insertion group had retinal reattachment (P = 1.000). Moreover, 16 eyes (69.6%) in the covering group and 17 eyes (68.0%) in the insertion group had their MHs sealed (P = 0.907). The best-corrected visual acuity (BCVA) at 12 months and the improvement in BCVA postoperatively in the two groups were not statistically significant (P = 0.543, 0.955). Logistic regression analysis showed that elongated AL (OR = 1.844, 95% CI 1.037–3.280, P = 0.037) and higher choroidal atrophy (OR = 2.986, 95% CI 1.011–8.821, P = 0.048) were risk factors affecting initial anatomical success. For extremely high-myopia MHRD with AL ≥ 30 mm, ILM flap covering and insertion can both effectively seal the MH and promote retinal reattachment, but the visual function improvement may still be limited. The longer the AL and the higher the choroidal atrophy, the greater is the risk of initial anatomical failure.

## Introduction

Macular hole retinal detachment (MHRD) is a severe complication of high myopia and one of the important causes of central vision loss, even blindness. The pathogenesis of MHRD is complex and may be related to vitreoretinal surface traction, incomplete posterior vitreous detachment (PVD), axial elongation, posterior scleral staphyloma, and chorioretinal atrophy^1,2^. Since Gonvers and Machemer^3^ reported pars planar vitrectomy (PPV) combined with gas tamponade for high-myopia MHRD in 1982, various surgical modalities have been reported, including pneumoretinopexy, PPV combined with gas or silicone oil (SO) or even high-density silicone oil (HDSO), internal limiting membrane (ILM) peeling with or without macular epiretinal membrane (ERM) removal, and macular buckling (MB). Despite these interventions, MHRD remains one of the most challenging types of RD to treat, with poor visual acuity recovery and the possibility of MH nonclosure or reopening and RD recurrence. In some patients, anatomical success may require multiple procedures.

As a classic surgical method for MHRD treatment in the last decade, PPV combined with ILM peeling can effectively eliminate the traction force in the retinal tangential direction. Several previous studies reported that the RD reattachment rate was 88–100%^4–6^, and the MH closure rate was 10–70%^7,8^. However, ILM removal cannot offset the anatomical imbalance caused by extension of ocular axial and posterior scleral grape swelling, leading to bare or flat-open RPE healing in these postoperative patients. Previous literature reported that the increase in axial length seemed to be related to the decrease in the MH closure rate, especially in patients with axial lengths greater than 30 mm^9,10^. When the axis of high myopia is seriously stretched to more than 30 mm, the posterior sclera is swollen, the retina is fragile, the choroid retina is atrophied, and the vitreoretinal interface is abnormal and sometimes accompanied by myopic traction macular retinal detachment. These characteristics may lead to a poor prognosis of MH surgery.

In 2010, Michalewska et al.^11^ first instructed the inverted ILM flap technique mainly used to treat large idiopathic MH (> 400 µm), with an MH closure rate being observed in 98% of cases, which promoted the application of this technique to treat high-myopia MHs with or without RD. Chen et al.^12^ further modified the ILM flap technique, in which the ILM flap is inserted invertedly into the MH rather than covering the MH to treat MHRD. Although the improvement in visual acuity compared with that in the ILM peeling group was not clear, all the MHs in the ILM insertion group were closed. These operations were based on the hypothesis that the inverted ILM flap in the MH was used as a tamponade and acted as a scaffold to correct the anatomical mismatch between the neurosensory retina RPE-choroidal-scleral complex^11,13^. However, surgical treatment can be complex in highly myopic eyes with extremely long axial lengths (AL ≥ 30 mm) or chorioretinal atrophy, and the long-term prognosis of these two procedures is still unclear. Thus, more data are needed to determine the efficacy of these two different types of inverted ILM flap techniques during vitrectomy for the treatment of MHRD in highly myopic eyes.

The purpose of this study was to compare the anatomical and functional outcomes of ILM flap covering and insertion techniques in the treatment of high-myopia MHRD with an axial length ≥ 30 mm and to further analyse the preoperative factors related to the success of initial surgical anatomy.

## Patients and methods

### Patients

This was a retrospective case series study consisting of patients with high-myopia MHRD who underwent 23-gauge PPV with an inverted ILM flap covering or insertion technique and SO tamponade in the Fundus Department of Wuhan Aier Eye Hospital of Wuhan University from January 2017 to January 2020. All cases were followed up for at least 12 months after the initial PPV operation. The investigations in this study adhered to the tenets of the Declaration of Helsinki. Approval of the study was obtained from the Ethics Committee of Aier Eye Hospital of Wuhan University (No.2021IRBKY914). Written informed consent for participation was obtained from all patients.

Inclusion criteria were as follows: (1) clinical presentation of a high-myopia macular hole associated with retinal detachment; (2) axial length ≥ 30 mm; (3) treatment with PPV combined with ILM flap covering or ILM flap insertion and SO tamponade; (4) a follow-up time more than 12 months after primary PPV, (5) having at least one SO removal operation during the follow-up period. The exclusion criteria were as follows: (1) presence of ocular disease or a systemic disease such as diabetes mellitus. (2) Previous ocular surgery, except for cataract surgery; (3) procedures using a transscleral approach, such as MB or scleral resectioning. (4) Other exclusion criteria including PVR C or above.

### Surgical procedures

All surgical procedures were performed by two experienced fundus surgeons (Jianhua Wu and Chao Feng). If the patient's lens opacity in phakic eyes affected the observation of the fundus, phacoemulsification surgery was performed first, and intraocular lens implantation was performed according to the intraoperative conditions. Then, a standard 23-gauge PPV (Constellation; Alcon Laboratories, Fort Worth, TX) was performed under retrobulbar or general anaesthesia on all patients. After removing the vitreous cortex of the central axis, the triamcinolone acetonide aqueous suspension was applied to visualize the posterior hyaloid to examine if posterior vitreous detachment (PVD) had fully occurred, and then the overlying ERM (if present) was removed by ocular forceps. Subsequently, 0.125% indocyanine green (ICG) solution was used to stain the ILM for 30 s. The ILM flap insertion technique and the ILM flap covering technique were chosen by the surgeon. Using the ILM flap insertion technique, approximately 1.5 to 2.0 disk diameter (DD) ILM was retained around the MH, and the residual ILM was peeled to the margins of the vascular arcade as soon as possible. The retained ILM flap was lifted to the edge of the hole with ILM forceps but was not peeled off. The flap was trimmed carefully with a vitreous cutter with low suction and then folded and inserted into the MH. The surgeons were cautious to avoid damaging the RPE and choroid. Using ILM flap covering technology, a semicircular ILM flap on the temporal or superior side of the macular hole with a diameter of 2.0–3.0 DD was retained, and the residual ILM was completely peeled to the margins of the vascular arcade. The remaining ILM flap was then detached from the retina by ILM forceps but remained attached on the edge of the MH. After trimming the ILM flap, the flap was massaged gently over the MH from all sides until it became inverted to cover the surface of the MH. After completion of the ILM inverted technique, an appropriate bubble amount of perfluorocarbon fluid (RT DECALIN 5 ml; Carl Zeiss Meditec AG, Jena, Germany) was injected intraoperatively to stabilize the ILM flap and make it more securely placed over or in the hole. If retinal detachment was localized within the arcade, fluid-air exchange was performed up to the staphyloma margin or slightly above the upper edge of the detached retina. If the detachment extended beyond the equator or a large amount of subretinal fluid remained, a drainage retinotomy hole was made with a vitreous cutter in the supratemporally detached retina region for drainage and fluid-air exchange. After fluid-air exchange, the surgeon confirmed whether the inserted or covered ILM flap was still in place. Laser photocoagulation was applied around the peripheral iatrogenic retinal breaks or lattice during the surgery to prevent subsequent RD. At the end of the surgical procedures, all operated eyes were subjected to tamponade with SO. All patients were instructed to maintain a face-down position for two weeks and then hold a lateral position until the SO was removed. The SO remained intraocular for at least 3 months, but was removed in advance if the intraocular pressure (IOP) increased out of control after surgery. The SO was removed surgically with flat scleral incision perfusion, and the SO was aspirated through a 23-gauge cannula.

### Ocular parameters

All patients underwent a complete ophthalmic examination before surgery and one day, 1 week, 3 months, 6 months and 12 months after surgery, including best-corrected visual acuity (BCVA), slit-lamp examination, and indirect ophthalmoscopy for fundal examination. The BCVA was evaluated using a Snellen chart as a functional outcome at baseline and 12 months after surgery. Axial length was measured by IOL Master (IOLMaster 500 or 700; Carl Zeiss Meditec AG, Jena, Germany) 1 week after primary surgery. Optical coherence tomography (SS-OCT, DRI OCT-1; Topcon Corp, Tokyo, Japan; and AngioVue Optovue, Fremont, CA) was performed at 1, 3, 6 and 12 months after the operation to evaluate the status of MH closure and recovery of the foveal microstructure, including the external limiting membrane (ELM) ellipsoid zone (EZ), gliosis, and hyperreflective bridging tissue (HBT). All parameters of foveal microstructures were assessed in a masked manner by two independent researchers (Tao Yan and Xiangyun Li), and consensus was reached with a third observer (Jianhua Wu) in case of disputes. The extent of the retinal detachment area was determined by panoramic scanning laser ophthalmoscope imaging (SLO; Optos 200Tx; Optos PLC, Dunfermline, Scotland) and divided into two categories accordingly: within or beyond the vascular arcade. The degree of chorioretinal atrophy was assessed according to SLO images one week after PPV surgery and referred to the classification of the Meta-analysis of Pathologic Myopia (META-PM) Study Group^14,15^: category 0, no myopic retinal lesion; category 1, tessellated fundus only; category 2, diffuse choroidal atrophy; category 3, patchy chorioretinal atrophy; and category 4, macular atrophy involving the fovea. Macular hole closure was defined as the absence of neurosensory defects in the macular fovea on OCT^16^. Retinal reattachment was defined as the complete absorption of subretinal fluid and the complete attachment of the neurosensory retina to the RPE. Initial anatomic success was defined as patients who underwent primary PPV and a single SO removal operation until the final follow-up period, with the retina remaining attached and the MH closed. Failure of the initial anatomy was defined as an unsuccessful retinal attachment or recurrent RD after primary surgery or retinal reattachment while the macular hole remained open.

We collected baseline demographic data, ocular characteristics, surgical procedures and outcomes, OCT and SLO findings, further surgery, and complications from the patients' medical records.

### Statistical analyses

For statistical analysis, the BCVA in the Snellen visual acuity ratio was converted to the logarithm of the minimum resolution angle (logMAR). Counting finger or hand movement vision was assigned as the equivalent Snellen acuity of 20/2000 (2.0 logMAR) or 20/20 000 (3.0 logMAR), respectively^17^. Statistical analysis was performed by SPSS version 25.0 (IBM Corporation, Armonk, NY). Continuous variables are expressed as the mean ± standard deviation (SD). The comparison of continuous variables, such as age, axial length, and preoperative and postoperative BCVA, was evaluated using an independent sample t test or Mann–Whitney U test. All categorical variables were expressed as the number of cases and percentages. Differences in the noncontinuous variables were evaluated using the chi-square test or Fisher’s exact test (if n < 5), as appropriate. Statistical significance was defined as P < 0.05. Binary logistic regression analysis was also used to investigate the associations between the initial anatomic success and several clinical factors, such as age, symptom duration time, axial length, posterior scleral staphyloma, META-PM grading, area of retinal detachment (within vs. beyond the staphyloma), ILM inversion technique (ILM covering vs. ILM insertion), combined phacoemulsification, duration of SO tamponade, and follow-up period from the initial surgery. Individual clinical factors were subjected to univariate analysis and were subsequently entered into the multivariate analysis by using an enter method if the P value was < 0.1. The criterion for retention in the multivariate model was P < 0.05.

## Results

### Comparison of baseline and surgery details

We retrospectively analysed the medical records of 48 patients with successful ILM inverted flap creation (23 eyes with ILM flap covering and 25 eyes with ILM flap insertion), including 5 males and 43 females, with an average age of (58.04 ± 11.46) years and an average axial length of (31.84 ± 1.35) mm. All patients underwent 23G PPV and SO tamponade. There were no significant differences in age, sex, axial length, preoperative lens status, duration of preoperative symptoms, retinal detachment range, posterior scleral staphyloma, or META-PM classification between the ILM covering group and the ILM insertion group (Table 1).

**Table 1:** Baseline characteristics of patients with MHRD who underwent inverted ILM flap covering technique or inverted ILM flap insertion technique.

	ILM flap covering (n = 23)	ILM flap insertion (n = 25)	Total (n = 48)	P
Age (year, mean ± SD)	56.70 ± 12.35	59.04 ± 10.55	57.92 ± 11.39	0.482
Sex (male/female), n (%)	2(8.7)/21(91.3)	3(12.0)/22(88.0)	5(10.4)/43(89.6)	1.000*
Axial length (mm, mean ± SD)	31.95 ± 1.19	31.74 ± 1.50	31.84 ± 1.35	0.600
Preoperative lens status (phakia/pseudophakia/ aphakia), n (%)	21(91.3)/2(8.7)/0(0)	21(84.0)/3(12.0)/1(4.0)	42(87.5)/5(10.4)/1(2.1)	1.000*
Duration of symptom (days, mean ± SD)	35.13 ± 31.37	29.76 ± 19.98	32.33 ± 25.92	0.876^†^
Extent of RD (within vascular arcade/beyond vascular arcade), n (%)	8(34.8)/15(65.2)	11(44.0)/14(56.0)	19(39.6)/29(60.4)	0.514
Posterior staphyloma (present/absent), n (%)	21(91.3)/2(8.7)	23(92.0)/2(8.0)	44(91.7)/4(8.3)	1.000*
Myopic maculopathy grading of the eye, n (%)				
Category 1	2(8.7)	1(4.0)	3(6.3)	0.567^†^
Category 2	10(43.5)	10(40.0)	20(41.7)	
Category 3	9(39.1)	12(48.0)	21(43.8)	
Category 4	2(8.7)	2(8.0)	4(8.3)	
Preoperative BCVA (logMAR unit, mean ± SD)	2.15 ± 0.55	2.08 ± 0.53	2.11 ± 0.54	0.628
Preoperative BCVA, Snellen visual acuity ratio	20/500–20/20,000	20/200–20/20,000	20/200- 20/20,000	–

*Fisher’s exact probability test; ^†^Mann–Whitney U test.

*logMAR* logarithm of the minimum angle of resolution, *RD* retinal detachment, *SD* standard deviation, *BCVA* best-corrected visual acuity.

There was no significant difference between the two groups in the PPV operation time, the number of eyes using heavy water, the presence of attached posterior hyaloid or ERM, the duration time of SO after PPV, whether cataract extraction was performed and the follow-up time (Table 2).

**Table 2:** Surgical procedure of patients with MHRD who underwent inverted ILM flap covering technique or inverted ILM flap insertion technique.

	ILM flap covering (n = 23)	ILM flap insertion (n = 25)	Total (n = 48)	P
Duration of PPV course (min, mean ± SD)	48.65 ± 10.18	49.80 ± 9.35	49.25 ± 9.67	0.686
Use of PFO, n (%)	11 (47.8)	13 (52.0)	24 (50.0)	0.773
Presence of attached posterior hyaloid or epiretinal membrane (present/absent), n (%)	10 (43.5)/13 (56.5)	11 (44.0)/14 (56.0)	21 (43.8)/27 (56.3)	0.971
Combined cataract surgery, non-PEA/PEA + IOL/PEA only, n (%)	5 (21.7)/6 (26.1)/12 (52.2)	10 (40)/8 (32.0)/7 (28.0)	15 (31.3)/14 (29.2)/19 (39.6)	0.203
Duration of SO before prim surg removal (months, mean ± SD)	4.65 ± 1.70	4.36 ± 1.66	4.50 ± 1.66	0.415^†^
Follow-up time (months, mean ± SD)	21.52 ± 4.11	22.40 ± 4.23	21.98 ± 4.15	0.470

*Fisher’s exact probability test.

^†^Mann–Whitney U test.

*PPV* pars planar vitrectomy, *PFO* perfluoro-n-octane, *PEA* phacoemulsification and aspiration, *IOL* intraocular lens, *SO* silicone oil.

### Comparison of anatomical and functional outcomes

The comparison of surgical outcomes between the two groups is shown in Table 3. After primary PPV, retinal attachment was achieved in both groups, and MH closure was observed in 17 eyes (73.9%) in the ILM flap covering group and 20 eyes (80.0%) in the ILM flap insertion group under the SO condition. There was no significant difference in the MH closure rate between the two groups (P = 0.616). After a single SO removal to the last follow-up period, there were 18 eyes (78.3%) in the ILM covering group and 20 eyes (80.0%) in the ILM insertion group with the retina remaining attached under non-SO conditions, and the macular holes remained closed in 16 eyes (69.6%) in the ILM covering group and 17 eyes (68.0%) in the ILM insertion group. The differences between the two groups in retinal attachment and MH closure rates were not statistically significant (P = 1.000 and 0.907, respectively). After SO removal, 5 eyes in each group experienced recurrent RD and underwent at least one or more additional surgeries to obtain retinal reattachment and macular hole repair. At the last follow-up, two eyes in the ILM covering group and one eyes in the ILM insertion group relied on SO tamponade to achieve retinal repositioning. The final MH closure was observed in 19 eyes (82.6%) in the ILM covering group, and 21 eyes (84.0%) in the ILM group, but there was no significant difference between the two groups (P = 1.000).

**Table 3:** Comparison of surgical outcomes.

	ILM flap covering (n = 23)	ILM flap insertion (n = 25)	Total (n = 48)	P
MH closure rate before SOR, n (%)	17 (73.9)	20 (80.0)	37 (77.1)	0.616
MH remained closed from PPV to the last follow-up, n (%)	16 (69.6)	17 (68.0)	33 (68.8)	0.907
Final MH closure rate (including after reoperation intervention), n (%)	19 (82.6)	21 (84.0)	40 (83.3)	1.000*
Retinal reattachment rate before SOR, n (%)	23 (100)	25 (100)	48 (100)	1.000*
Retina remains attached from PPV to the last follow-up, n (%)	18 (78.3)	20 (80.0)	38 (79.2)	1.000*
Final Retinal reattachment rate (including eyes filled with SO), n (%)	23 (100)	25 (100)	48 (100)	1.000*
Retina maintained reattachment under SO at last follow-up, n (%)	2 (8.7)	1 (4.0)	3 (6.3)	0.601*
Restoration of EZ at postoperative 12 months, n (%)	0	0	0	> 0.999
Restoration of ELM at postoperative 12 months, n (%)	0	0	0	> 0.999
Presence of HBT, n (%)	5 (21.7)	7 (28.0)	12 (25.0)	0.617
Presence of gliosis, n (%)	4 (17.4)	3 (12.0)	7 (14.6)	0.696*
Postoperative BCVA at 12 months (logMAR, mean ± SD)	1.98 ± 0.46	1.90 ± 0.42	1.94 ± 0.44	0.543
Final lens status (phakia/pseudophakia/ aphakia) n (%)	3(13.0)/8(34.8)/12(52.2)	6(24.0)/11(44.0)/8(32.0)	9(18.8)/19(39.6)/20(41.7)	0.386*

*Fisher’s exact probability test.

*MH* macular hole, *SOR* silicone oil removal, *PPV* pars planar vitrectomy, *SO* silicone oil, *EZ* ellipsoid zone, *ELM* external limiting membrane, *HBT* hyperreflective bridging tissue, *BCVA* best-corrected visual acuity.

**Figure 1:**
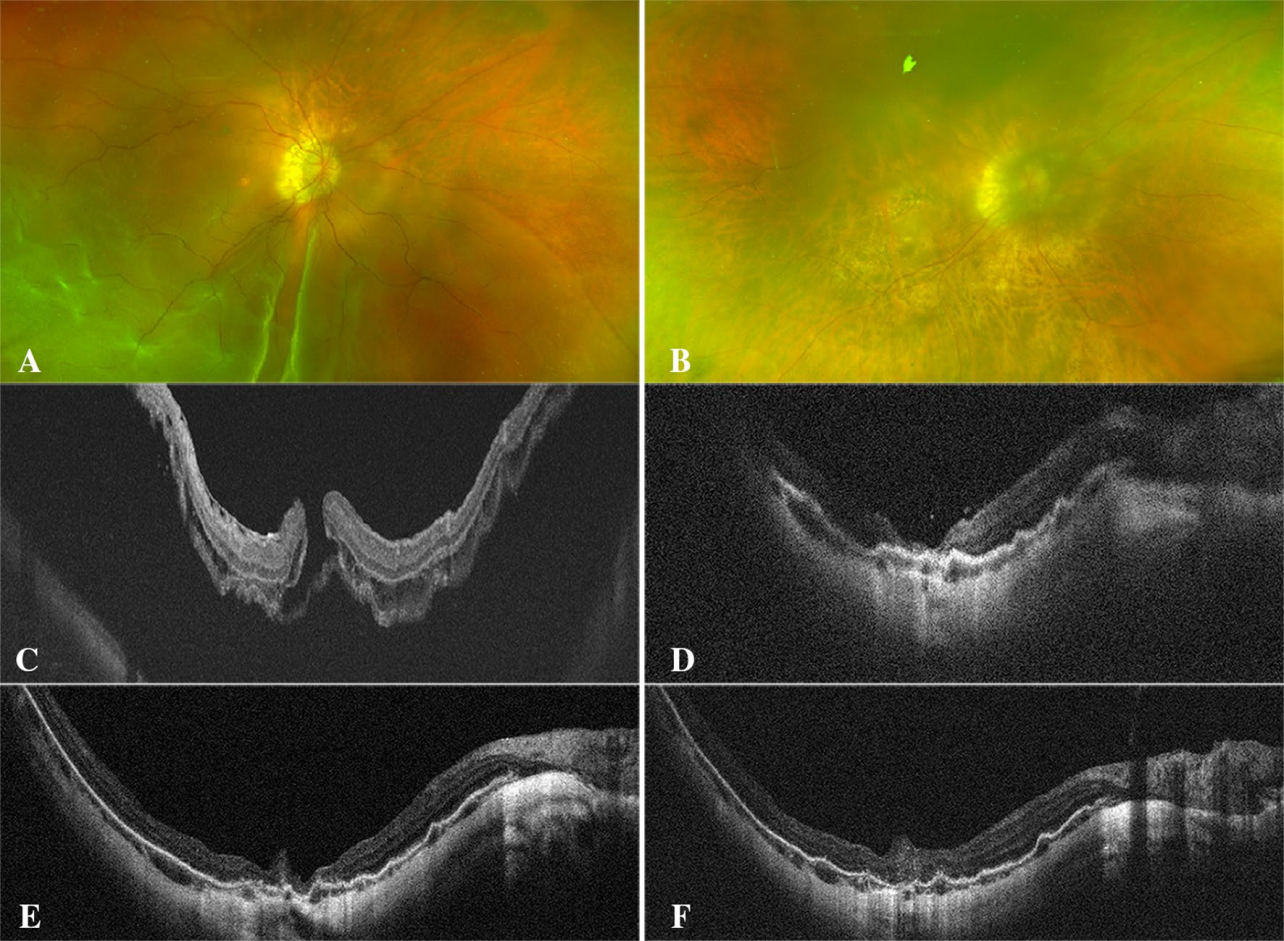
A 57-year-old woman with high-myopia macular hole retinal detachment (axial length, 30.71 mm; META-PM classification, C1) underwent PPV combined with ILM flap insertion (**A**–**C**). One week after the operation, the OCT image presented a small amount of medium and high reflectors (the filled ILM flap) in the macular hole (**D**). One month after the operation, a tissue equivalent to the surrounding retinal reflector increased (gliosis), which sealed the hole together with the medium and high tissue reflectors (**E**). Twelve months after the operation, gliosis and diffuse distribution of medium and high reflectance were observed (**F**).

**Figure 2:**
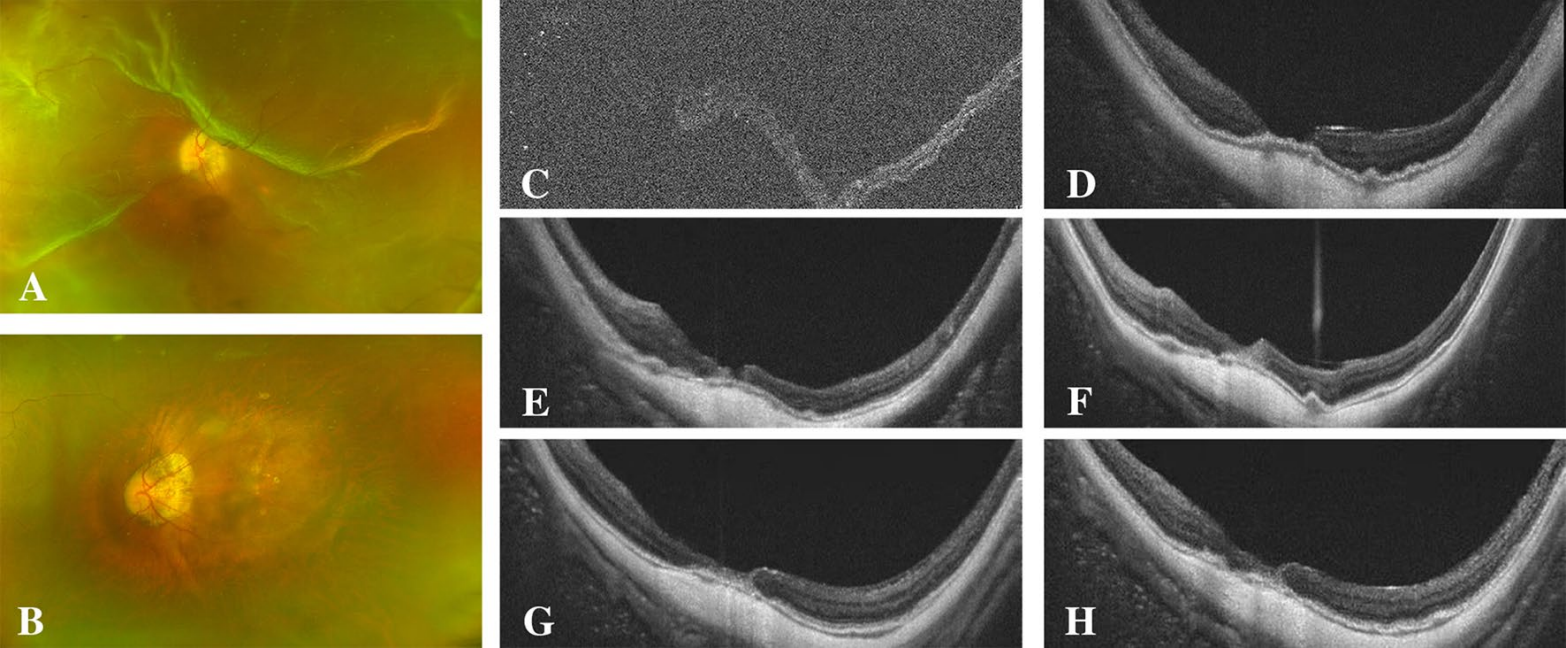
A 42-year-old man with high-myopia macular hole retinal detachment (axial length, 32.76 mm; META-PM classification, C2) underwent PPV combined with ILM flap insertion (**A**–**C**). One week after surgery, the macular hole was not closed, and a "W" shape was observed (**D**). One month after surgery, high-reflector tissue was found in the macula, and the MH was still partially open (**E**). The hole was closed by high-reflector tissue 3 months after surgery (**F**). Hyperreflective bridging tissue with gliosis at 6 **(G) **and 12 months postoperatively (**H**).

**Figure 3:**
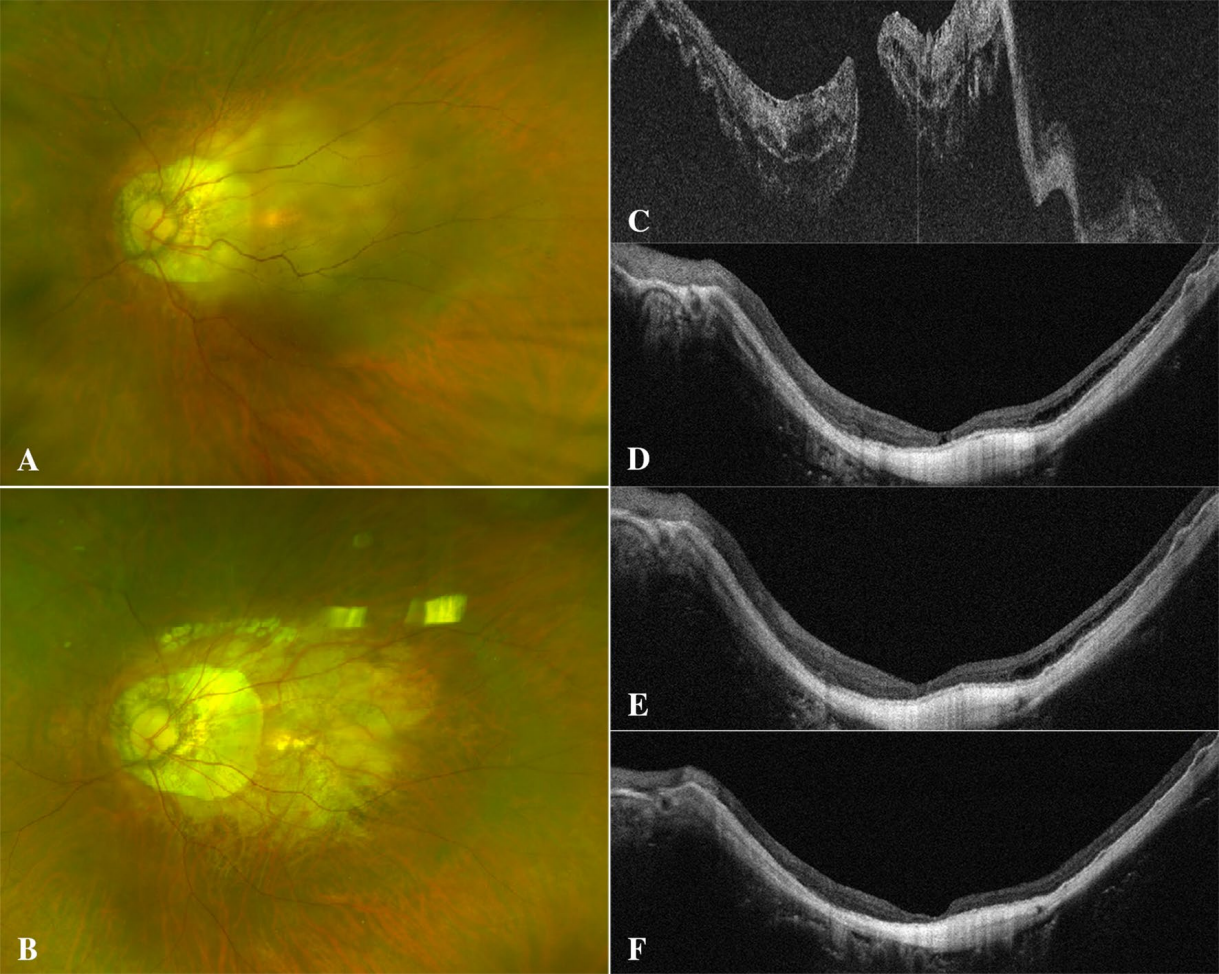
A 66-year-old woman with high-myopia macular hole retinal detachment (axial length, 32.46 mm; META-PM classification, C3) underwent PPV combined with ILM flap covering (**A**–**C**). One week after surgery, an inverted ILM covered the surface of the macular hole, and nasal subretinal fluid was present (**D**). One month after the operation, nonretinal tissue closed the hole, and there was no significant change in the nasal subretinal fluid (**E**). Six months after the operation, the reflection of nonretinal tissue in the macular hole increased slightly, and the nasal subretinal fluid was absorbed (**F**), but the EZ and ELM failed to recover.

**Figure 4:**
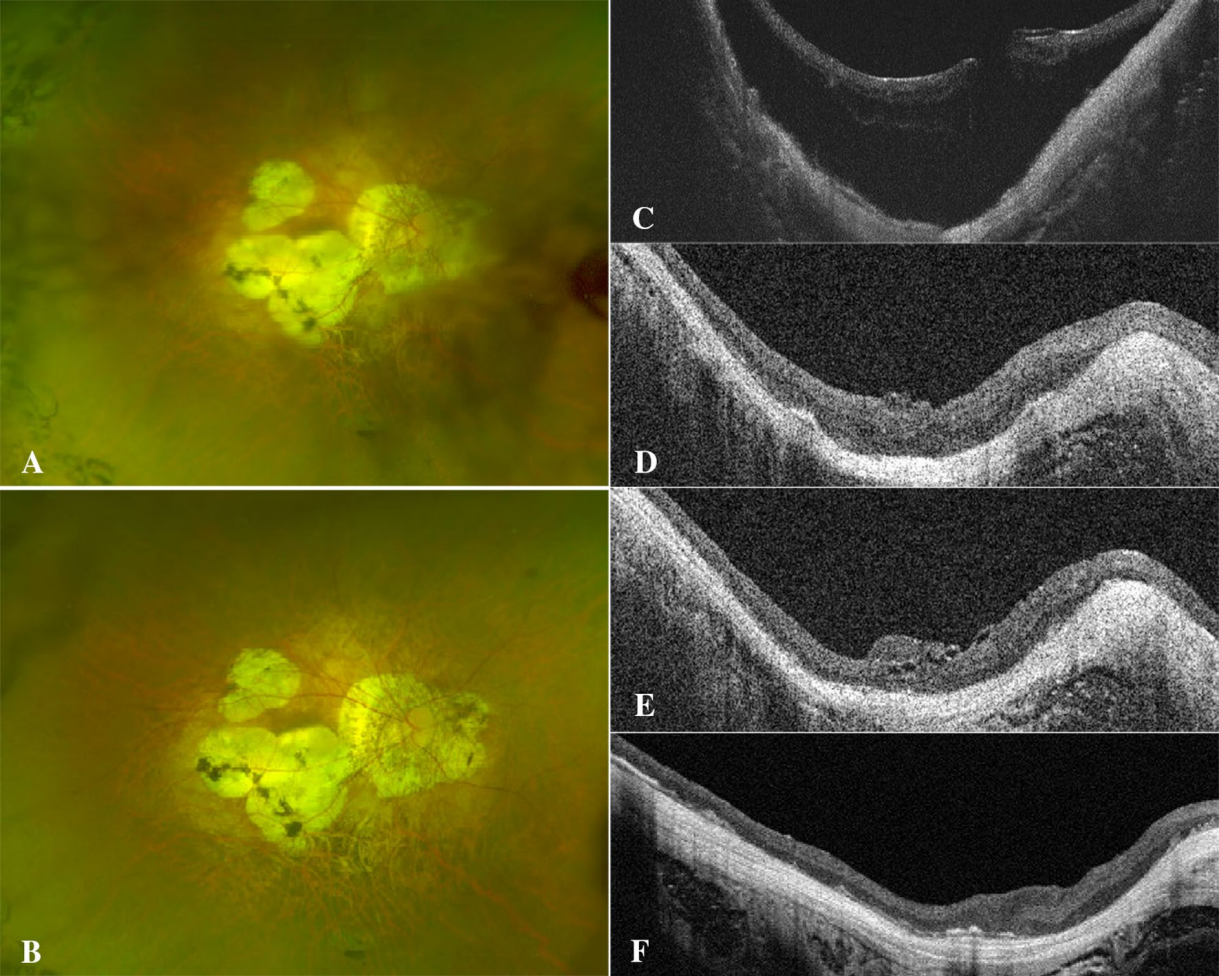
A 53-year-old man with high-myopia macular hole retinal detachment (axial length, 31.33 mm; META-PM classification, C4) underwent PPV combined with ILM flap covering (**A**–**C**). At one month after PPV combined with ILM flap covering, the macular hole had already closed, and the inverted ILM flap was still visible on the surface of the macular hole (**D**); the ILM flap was seen on the closed MH surface six months after the operation, accompanied by excessive glial proliferation (**E**). A large area of nonretinal tissue was seen in the fovea 12 months after the operation, the EZ and ELM interruption failed to recover (**F**).

The postoperative BCVA of the ILM covering group at 12 months after surgery was (1.98 ± 0.46) logMAR and (1.90 ± 0.42) logMAR in the ILM insertion group, which was improved by (0.17 ± 0.60) logMAR and (0.18 ± 0.67) logMAR, respectively, and the difference was not statistically significant (paired T test, P = 0.189, 0.190). Nevertheless, the comparison of BCVA between the two groups at 12 months showed no statistical significance (independent sample T test, P = 0.543). None of the eyes had EZ or ELM recovery at 12 months postoperatively in either group. In addition, there were no significant differences in the number of eyes with HBT and gliosis between the two groups (P = 0.617 and 0.696 respectively) (Figure. 1, 2,3, 4).

### Logistic regression analysis associated with initial anatomic success

The results of univariate analysis that was conducted to detect the difference between the eyes with initial anatomic success and those with initial failure are summarized in Table 4. A total of 15 eyes (31.3%) experienced initial surgical anatomic failure after PPV + SO tamponade or subsequent single SO removal surgery. Five eyes failed because MH was unclosed after primary surgery, but the retina remained attached even with SO removal. The other ten eyes were caused by MH reopening with recurrence of RD after SO was removed.

**Table 4:** Univariate analysis between baseline demographics, surgical treatments, and anatomical outcome after primary PPV and SO tamponade.

	Initial success (n = 33)	Initial failure (n = 15)	Total (n = 48)	P
Age (year, mean ± SD)	57.91 ± 11.92	57.93 ± 10.76	57.92 ± 11.39	0.995
Sex (male/female), n (%)	3 (9.1)/30 (90.9)	3 (20.0)/12 (80.0)	6 (12.5)/42 (87.5)	0.360*
Axial length (mm, mean ± SD)	31.51 ± 1.14	32.56 ± 1.52	31.84 ± 1.35	0.011
Preoperative lens status (phakia/pseudophakia/ aphakia), n (%)	30 (90.9)/3 (9.1)/0 (0)	12 (80.0) /2 (13.3)/1 (6.7)	42 (87.5)/5 (10.4)/1 (2.1)	0.239^†^
Duration of symptom (days, mean ± SD)	30.27 ± 24.87	36.87 ± 28.44	32.33 ± 25.92	0.362^†^
Extent of RD (within vascular arcade/beyond vascular arcade), n (%)	11 (33.3)/22 (66.7)	8 (53.3)/7 (46.7)	19 (39.6)/29 (60.4)	0.189
Posterior staphyloma (present/absent), n (%)	31 (93.9)/2 (6.1)	13 (86.7)/2 (13.3)	44 (91.7)/4 (8.3)	0.579*
**Myopic maculopathy grading of the eye,** **n **(**%)**				
Category 1	3 (9.1)	0 (0)	3 (6.3)	0.038^†^
Category 2	16 (48.5)	4 (26.7)	20 (20)	
Category 3	12 (36.4)	9 (60)	21 (43.8)	
Category 4	2 (6.1)	2 (13.3)	4 (8.3)	
Presence of attached posterior hyaloid or epiretinal membrane (present/absent), n (%)	15 (45.5)/18 (54.5)	6 (40.0)/9 (60.0)	21 (43.8)/27 (56.3)	0.724
Combined ILM covering/insertion, n (%)	16 (48.5)/17 (51.5)	7 (46.7)/8 (53.3)	23 (47.9)/25 (52.1)	0.907
Combined cataract surgery, non PEA/PEA + IOL/PEA only, n (%)	10 (30.3)/12 (36.4)/11 (33.3)	5 (33.3)/3 (20.0)/7 (46.7)	15 (31.3)/15 (31.3)/18 (37.5)	0.671^†^
Duration of SO before prim surg removal (months, mean ± SD)	4.21 ± 1.56	5.13 ± 1.77	4.50 ± 1.66	0.075

*RD* retinal detachment, *PEA* phacoemulsification and aspiration, *IOL* intraocular lens, *SO* silicone oil.

*Fisher’s exact probability test.

^†^Mann–Whitney U test.

Univariate analysis showed that axial length, META-PM grade and duration of initial SO tamponade were the related preoperative factors with a P value < 0.1. The axial length of the eyes with initial anatomical failure (32.56 ± 1.52) mm was longer than that of eyes with successful (31.51 ± 1.14) mm, with significant differences (P = 0.011). The META-PM grade of the eyes with initial success was lower than that of failed eyes, with a significant difference (P = 0.038) (Table 4). Subsequently, multivariate analysis was performed for these three factors, which confirmed that a prolonged axial length (odds ratio [OR] = 1.844, 95% confidence interval [95% CI] 1.037 to 3.280, P = 0.037) and a high grade of META-PM (OR = 2.986, 95% CI 1.011 to 8.821, P = 0.048) were significantly associated with initial anatomical failure (Table 5).

**Table 5:** Logistic regression model summary for initial anatomical success after primary PPV and SO tamponade.

Independent variable	Odds ratio	95% confidence interval	P value
Axial length	1.844	1.037–3.280	0.037
Myopic maculopathy grading of the eyes	2.986	1.011–8.821	0.048
Combined ILM flap surgery (covering vs insertion)	1.317	0.310–5.595	0.709
Duration of silicone oil before prim surg removal	1.445	0.948- 2.203	0.087

### Postoperative complications

**Figure 5:**
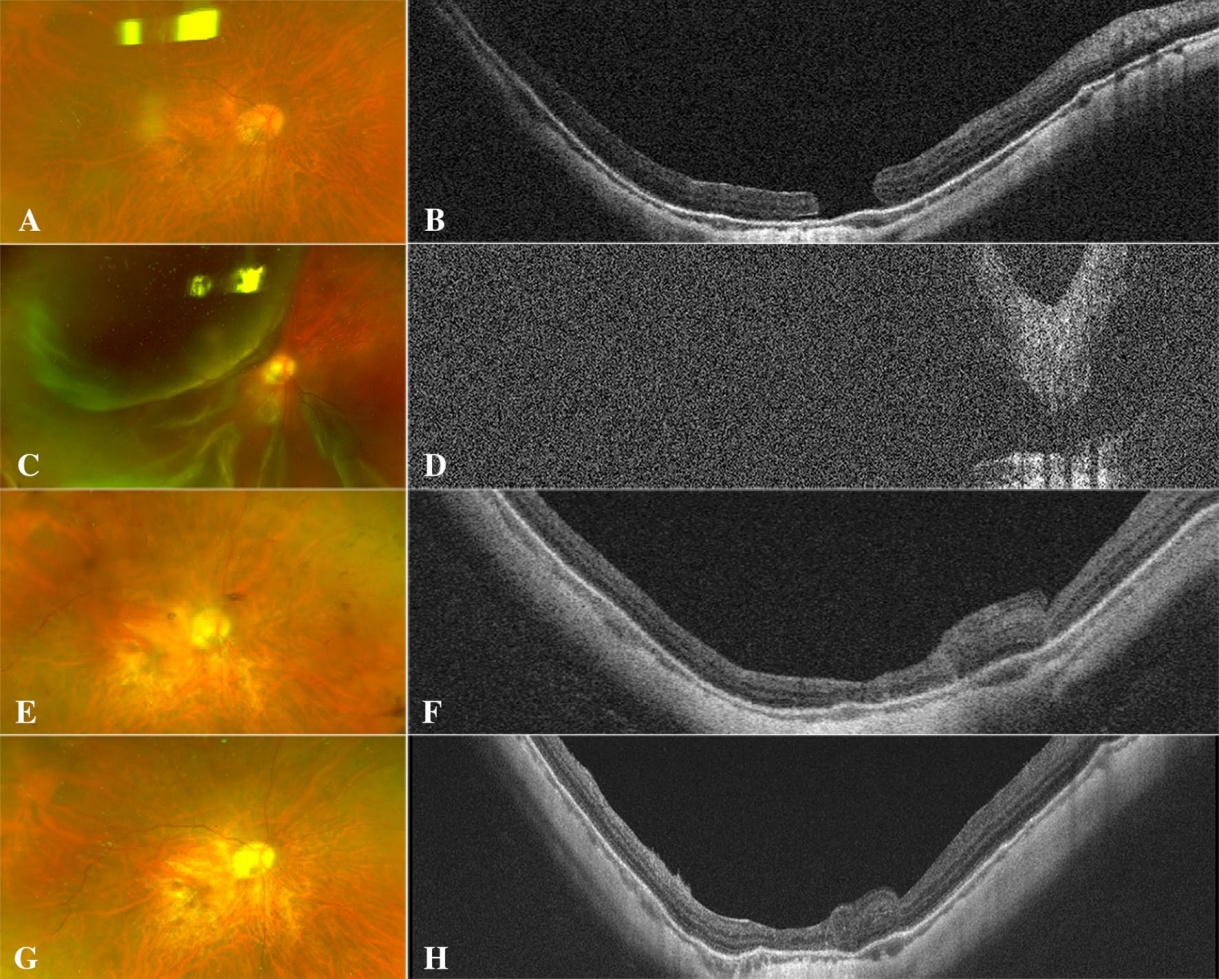
A case of recurrent high-myopia macular hole retinal detachment treated by autologous neurosensory retinal transplantation (ANRT). A 61-year-old woman with high-myopia macular hole retinal detachment (axial length, 31.32 mm; META-PM classification, C2) had retinal reattachment achieved after PPV and ILM flap covering combined with silicone oil tamponade (**A**); however, the macular hole was not closed under the silicone oil condition (**B**). One month after silicone oil removal, the macular hole reopened, and retinal detachment recurred (**C**,**D**). Subsequently, a second operation was performed with ANRT and silicone oil refilling. One week after reoperation, the retina was reattached under silicone oil conditions (**E**). The OCT image shows the structural layer of the retinal graft overlaying the macular hole (**F**). Three months after ANRT, the retina was reattached (**G**), and the graft fused with the surrounding retinal tissue and sealed the macular hole successfully (**H**).

After the SO was removed, retinal redetachment caused by MH reopening occurred in 5 eyes of both groups (Table 6). A total of 5 eyes (50%) underwent retinal reattachment surgery combined with autologous neurosensory retinal transplantation (ANRT) and a second SO tamponade after recurrence (Figure. 5). Among them, four eyes (80%) obtained complete anatomic success (retinal reattachment with MH closure), and one eye (Case 8) retina reattached under the SO condition, while the MH remained open. Another 2 cases (20%) underwent ERM removal, and one eye (10%) underwent extended ILM peeling during reoperation. Among them, one case succeeded in reattaching the retina under SO conditions, but the macular hole remained open. The remaining 2 cases (20%) of the ten recurrent cases underwent macular photocoagulation + SO or air filling. Among them, the macular hole of case 3 was still open under SO conditions. However, this patient was advanced in age and refused to have the silicone oil removed again.

**Table 6:** Details of 10 cases of recurrent retinal detachment due to reopening of MH.

Cases	Age (years)	Sex (male/female)	Axial length (mm)	META-PM classification	Primary PPV combine ILM inverted flap (covering/insertion)	MH status under SO (open/closed)	Diameter of MH under SO (um)	Filling time of SO (months)	Time of RD recurrence	The main intervention of reoperation	Refilling time of SO (months)	Follow up time (months)	Final status of MH	Final status of retina
1	61	Female	31.23	2	Covering	Open	445	6	1 month after SOR	ANRT + SO	3	18	Closed	Attached
2	49	Male	31.17	2	Insertion	Closed	–	4	3 months after SOR	ANRT + SO	4	24	Closed	Attached
3	73	Female	31.14	3	Covering	Open	1872	4	When SOR	Fluid-air exchange + Photocoagulation around foveal + SO	None SOR	28	Open	Under SO
4	66	Female	32.07	3	Insertion	Open	1646	3	1 month after SOR	ANRT + SO	6	24	Closed	Attached
5	33	Female	31.69	3	Covering	Open	1437	8	When SOR	ANRT + SO	3	18	Closed	Attached
6	56	Female	34.16	3	Covering	Open	638	3	1 week after SOR	Fluid-air exchange + ERM removal + SO	None SOR	20	Open	Attached under SO
7	48	Female	34.05	3	Covering	Closed	–	8	When SOR	Fluid-air exchange + Extended ILM Peeling + Massage	–	24	Closed	Attached
8	50	Female	33.23	2	Insertion	Open	1463	6	3 months after SOR	PVR Removal + ANRT + SO	None SOR	25	Open	Attached under SO
9	53	Male	35.46	3	Insertion	Closed	–	4	4 months after SOR	ERM Removal + serum + air	–	24	Closed	Attached
10	72	Female	30.96	3	Insertion	Closed	–	7	1 week after SOR	Fluid-air Exchange + Photocoagulation around foveal	–	28	Closed	Attached

*META-PM* meta-analysis of pathologic myopia, *PPV* pars planar vitrectomy, *ILM* internal limiting membrane, *MH* macular hole, *SO* silicone oil, *RD* retinal detachment, *SOR* silicone oil removal, *ANRT* autologous neurosensory retinal transplantation, *ERM* epiretinal membrane.

## Discussion

High-myopia MHRD is one of the severe diseases that threaten vision. In the past decade, PPV combined with ILM peeling, with or without ERM removal, intraocular gas or SO tamponade, has been regarded as one of the most effective treatments for MHRD. Although the retinal reattachment rate increased, the closure rate of MH was still not very satisfactory. In a study by Fang et al.^6^, 24 of 30 eyes with successfully reattached retinas were examined by OCT; only 46% of the eyes had a normal concave macular structure, and 54% of people had a central defect in the retina, even though the edges around the MH were firmly attached to the underlying RPE. Ikuno et al.^18^ observed by OCT that 44% of eyes had MH closure, and the remaining 56% had persistent MH. Theoretically, peeling the ILM can remove the cortical vitreous, release tangential traction, relax the retina, and promote MH closure and retinal reduction in patients with MHRD. However, for high- myopia MHs or MHRD with an axial length of more than 30 mm, peeling the ILM makes it insufficient to eliminate the elongated axial length and retinal tension, and it is challenging to obtain MH closure and retinal reattachment. In addition, due to severe posterior scleral staphyloma, chorioretinal atrophy, and an abnormally thin retina, the operation is more complicated to identify preretinal structures such as PVD, ERM, and ILM, which may lead to surgical failure. A retrospective study by Suda et al.^9^ showed that elongation of the axial length was a risk factor for MH closure, and in highly myopic eyes with axial lengths of 30.0 mm or more, the initial and overall success rates were both 0%.

The inverted ILM flap technique was first proposed by Michalewska et al.^11^ and is mainly used to treat large idiopathic MH (> 400 µm). Its main operation is to peel the ILM while retaining part of the ILM tissue at the edge of the MH and then fold it back and cover it on the MH after trimming. They hypothesized that the inverted ILM flap could provide a scaffold for the proliferation of glial cells and eventually seal the MH, thereby enhancing the closure of the MH. Kuriyama et al.^13^ extended this procedure to high myopia with MH and MHRD and achieved a high macular hole closure rate and successful retinal reattachment. Chen et al.^12,19^ further modified the technique of ILM flap, in which the ILM flap is inserted invertedly into the MH rather than covering the MH to treat MHRD. Although the improvement in visual acuity compared with that in the ILM peeling group was not certain, all the MHs in the ILM insertion group achieved decent MH closure. A growing number of studies have used ILM flap technology to achieve exciting results^8,20–26^. For high myopia, ILM flap technology has unique advantages: on the one hand, ILM peeling can not only completely remove the cortical vitreous but can also release vitreous macular traction, and stretch the retina. More importantly, covered or inserted ILM flaps can be used as scaffolds to stimulate the proliferation of glial cells, compensate for the anatomical imbalance caused by axial lengthening of the eye, and promote the closure of MHs and retinal reattachment. A retrospective study by Olenik et al.^27^ reported the efficacy of ILM flap covering technology in high myopia MH without RD with an axial length greater than 30 mm. One month after the operation, 33 cases in their study were closed entirely (100%)—with only 2 cases of MH reopening later. A few cases of extremely high myopia with an average ocular axial length of more than 30 mm with the ILM flap inverted technique were included in several previous studies, which yielded better anatomic and functional outcomes^23,28–30^. However, until now, the number of extremely high myopia MHRD patients included in these studies was still very limited, and there was a lack of comparative studies on the efficacy of these two techniques in such patients.

Recently, a retrospective study by Zhu et al.^22^ compared anatomical and functional results of MHRD by using an ILM covering or insertion ILM flap after vitrectomy at 12 months; the MH closure rate in the ILM flap coverage group seemed to be higher than that in the ILM insertion group, although the difference was not statistically significant (95% vs. 73%, p = 0.059). In our study, the ILM coverage group and ILM insertion group were equally effective in treating extremely high myopia MHRD, and both groups achieved good anatomical results. There was no significant difference between the two groups in either the MH closure rate under the SO condition (ILM covering group 73.9% vs. ILM insertion group 80.0%, P = 0.616) or the final MH closure rate (ILM covering group 82.6% vs. ILM insertion group 84.0%, P = 1.000). The MH closure rate (69.6% and 68.0%, respectively) and retinal reattachment rate (78.3 and 80%, P = 1.00) were similar in the two groups from PPV and SO removal to the last follow-up. After primary PPV and a single SO removal until the last follow-up period, the macular hole closure rate (69.6% and 68.0%, respectively) and retinal reattachment rate (78.3% and 80.0%, P = 1.00) between the two groups were similar. In the subgroup analysis of this study, it was found that the initial MH closure rate and initial retinal reattachment rate in both the ILM covering and ILM insertion groups seemed to be lower than those reported in similar studies. The reason may be related to the longer ocular axial length of the patients we included, which was more severe than that in previous studies. The longer the ocular axis is, the greater the reverse traction of the sclera to the retina. Reattachment needs to overcome the reverse growth, and the extension of the retinal tissue is not enough to compensate for the backwards extension of the sclera and affect the closure of the macular hole^4,31,32^. Unsurprisingly, multivariate logistic regression analysis also further confirmed the clinical significance of ocular axis extension for the anatomical success of high MHRD; that is, for every 1 mm increase in ocular axial compression, the risk of failure of the initial anatomy of the retina was increased by 1.844 times (OR = 1.844, 95% CI 1.037–3.280, P = 0.037). In addition, logistic regression analysis also revealed that the pattern of ILM inverted flaps was not correlated with the success of MHRD anatomy (OR = 1.317, 95% CI 0.310–5.595, P = 0.709).

Another important risk factor for the success of the initial anatomy in MHRD eyes is META-PM grading. The higher the META-PM grade was, the more severe the choroid retinal atrophy was and the higher the incidence of retinal anatomical failure was (OR = 2.986, 95% CI 1.011–8.821, P = 0.048). On the one hand, the RPE and choroid atrophy and thinning and the choroidal blood supply decrease in eyes with an axial length of more than 30 mm in highly myopic eyes, which weakens the adhesion between the neurosensory retina and the posterior pole RPE layer and choroid, leading to difficulty in retinal reattachment. On the other hand, in eyes with high myopia, the ILM is very brittle and firmly adheres to other retinal layers, and due to significant choroidal atrophy, it is often difficult to identify or fully relax its traction on the hole during surgery^33^, which may easily lead to reopening of the MH after surgery.

In this study, we included all patients with silicone oil-filled eyes. For extremely high-myopia MHRD with PPV combined with the ILM flap inverted technique, SO was selected as a tamponade, which may have the following advantages. First, since viscous subretinal fluid and an uneven posterior pole structure usually accompanied each other in MHRD eyes, it is often difficult to fully drain the subretinal fluid during the operation. With the help of the SO, this may be conducive to the rapid absorption of residual subretinal fluid by RPE, obviating any manipulation of the macula and reducing the risk of damage to the MH margin to prevent displacement of the inverted ILM flap after the operation. Second, the hyperopic shift because of SO tamponade in phakic, aphakic, or pseudophakic eyes reduced myopia, yielding faster rehabilitation with the rapid postoperative restoration of visual function. Third, SO can not only act as a tamponade facilitating observation of the ILM flap status in the early stage after the operation but can also act as an inducer and scaffold for proliferative glial closure of the causative MH^4^. Some studies have also pointed out that the duration of SO filling is a relevant preoperative factor affecting the retinal reattachment rate in patients with MHRD. However, we did not find an effect of this factor on initial anatomic success in our study (OR = 1.445, 95% CI 0.948–2.203, P = 0.087).

The recovery of visual function was contrary to expectations, and neither the ILM flap covering technique nor the ILM flap insertion technique significantly improved the postoperative visual acuity even after MH closure. We also found no significant difference in the BCVA between the two groups postoperatively. Michalewska et al.^11^ reported that inverted ILM flap technology not only improves the anatomical success rate for large MHs but also improves the functional outcomes after vitrectomy. They hypothesized that inverted ILM flap technology induces glial cell proliferation and provides a new position environment directly close to the fovea for photoreceptors to improve postoperative visual acuity. Zhu et al.^22^ noted that ILM insertions may aggregate in the central fovea, thus interfering with the migration of Müller cells and damaging the recovery of photoreceptors. Meanwhile, the contact between ICG-stained ILMs and subretinal tissue during ILM insertion may lead to toxicity of the RPE and choroid. Therefore, compared with ILM insertion technology, ILM coverage technology has significantly improved the postoperative BCVA of MHRD patients. Their multivariate analysis further found that preoperative BCVA and ILM coverage were independent prognostic factors for the visual recovery of MHRD patients. However, it should be noted that not all the subjects included in their study highly were myopic patients with a long ocular axis. This may explain the differences in our results. First, in extremely high myopia with a long axial length and prominent posterior scleral staphyloma, a MH often occurs without subjective symptoms and remains undetected for a long time until RD occurs; therefore, irreversible damage to the fovea is more likely to have occurred before primary vitrectomy. Second, there is usually extensive chorioretinal atrophy, and the structure of the photoreceptor layer in the fovea may have been destroyed before surgery; even if the MH is successfully closed after surgery, the damage to the outer layer of the retina may still be irreversible postoperatively. Third, the lack of homogeneity between the ILM flap and the surrounding nerve structure in the inverted technology does not help transmit nerve stimulation, and therefore, the improvement in BCVA is likely limited^34^. Therefore, we believe that in long-axis high-myopia MHRD, macular hole closure using internal limiting membrane covering or insertion technology is expected to prevent retinal redetachment in the future. However, the improvement in visual function, especially the recovery of central vision, is seemingly limited.

Although both the ILM covering and insertion groups achieved a high initial anatomic success rate, the restoration of the foveal microstructure seemed quite unsatisfactory. None of the eyes exhibited EZ and/or ELM recovery at 12 months postoperatively in either group in the present study (Figure. 1, 2, 3, 4). Hayashi et al.^25^ performed PPV and inverted ILM flap techniques on highly myopic eyes with MHRD, and ELM and EZ restoration on OCT was found in only 17% (1/6) of the eyes after surgery. They believed that although the retina may have been reattached after reversing the ILM flap technique, the fovea may have been damaged beyond repair in high myopia. Moreover, another study revealed that the recovery of foveal microstructures may take more than one year or even longer. Our findings further support Hayahi's view that the foveal microstructure might be destroyed beyond recovery despite MH closure in highly myopic eyes with MHRD; however, a more extended follow-up period is still necessary.

For postoperative complications, we mainly focused on reopening the MH and the recurrence of RD. Since the vitreous and ILM have been removed, the operation is becoming more challenging. Currently, some alternative techniques have been reported for refractory macular holes, such as autologous ILM flap transplantation, ANRT, human amniotic membrane (hAM) plug, and so on. Several studies that use the hAM plug have been proven to be effective in repairing large^35^ or failed macular holes^36,37^ and high-myopic macular holes associated with retinal detachment^38^, with a 93.8% closure rate after one surgical intervention^35^. However, we have not yet carried out these technologies for legal reasons. In this study, we had five eyes that underwent ANRT and SO refilling surgery, and 80% of cases (4/5) were finally anatomically reattached successfully (RD reattachment and MH closure), which is consistent with that in our previous report^39^. Recently, an international multicenter study also reported that ANRT could obtain a high reclosure rate of 87.8% in the treatment of an unclosed high-myopia MH after PPV combined with ILM peeling^40^. Therefore, our study also provides some evidence for the effectiveness of treatment for recurrent MHRD due to MH reopening and ILM removal.

Of course, our study still has several limitations. First, it was a retrospective observational study, and the ILM flap technique was not a random choice but depended on the surgeon's selection according to the conditions and severity of individual cases, which may have been biased. Second, since it was difficult to measure the MH diameter in retinal detachment situations, the influence of this factor on the research results was not considered in the present study. Third, in the analysis of visual function outcomes, we only considered the BCVA at 12 months postoperatively, as well as other functional tests, such as visual field, electrophysiological and microperimetry, which should also be comprehensively evaluated further. Finally, the number of cases was relatively small, and the follow-up period was short. Future studies with a prospective design, larger sample size, longer follow-up time, more scientific assessment of visual acuity, and more specific examinations are needed to support our conclusions.

## Conclusion

In conclusion, for MHRD patients with an axial length ≥ 30 mm, the ILM flap covering or insertion technique can effectively seal the macular hole and promote retinal reattachment, but the improvement in visual function is still limited. We also found that a longer axial length and META-PM grade were important risk factors affecting the success of the initial anatomy in these patients. Since the number of study eyes analysed is still small and the study is retrospective, these findings should be interpreted cautiously. Prospective studies with a larger sample size and a more extended follow-up period are needed to determine the difference in visual results between the two techniques.

## Data Availability

The data used to support the findings of this study are available from the corresponding author upon request.
